# Contrast-Enhanced Ultrasound (CEUS) for Follow-Up of Bosniak 2F Complex Renal Cystic Lesions—A 12-Year Retrospective Study in a Specialized European Center

**DOI:** 10.3390/cancers12082170

**Published:** 2020-08-04

**Authors:** Johannes Rübenthaler, Saša Čečatka, Matthias Frank Froelich, Matthias Stechele, Constantin Marschner, Bastian Oliver Sabel, Florian Bogner, Moritz Ludwig Schnitzer, Daniel Overhoff, Nils Große Hokamp, Michael Staehler, Vincent Schwarze, Dirk-André Clevert

**Affiliations:** 1 Department of Radiology, University Hospital LMU, Marchioninistrasse 15, 81377 Munich, Germany; Johannes.Ruebenthaler@med.uni-muenchen.de (J.R.); sasa.cecatka@googlemail.com (S.Č.); Matthias.Stechele@med.uni-muenchen.de (M.S.); constantin.marschner@med.uni-muenchen.de (C.M.); bastian.sabel@med.uni-muenchen.de (B.O.S.); fbogner@uni-koeln.de (F.B.); moritz.schnitzer14@gmail.com (M.L.S.); dirk.clevert@med.uni-muenchen.de (D.-A.C.); 2 Institute of Clinical Radiology and Nuclear Medicine, University Medical Center Mannheim, Theodor-Kutzer-Ufer 1–3, 68167 Mannheim, Germany; Matthias.Froelich@medma.uni-heidelberg.de (M.F.F.); daniel.overhoff@umm.de (D.O.); 3 Institute for Diagnostic and Interventional Radiology, Faculty of Medicine and University Hospital Cologne, University Cologne, Kerpener Str. 62, 50937 Cologne, Germany; nils.grosse-hokamp@uk-koeln.de; 4 Department of Urology, University Hospital LMU, Marchioninistrasse 15, 81377 Munich, Germany; michael.staehler@med.uni-muenchen.de

**Keywords:** Bosniak 2F, renal cell carcinoma, RCC, contrast-enhanced ultrasound, CEUS, follow-up, urology

## Abstract

Bosniak 2F renal cystic lesions feature morphologic characteristics between Bosniak I and III categories, the majority of which remain benign. However, a minor part of Bosniak 2F lesions may progress to malignancy. The purpose of this study was to assess Bosniak 2F cystic lesions during follow-up examinations by CEUS. One-hundred-and-twelve out of 364 patients with Bosniak 2F lesions underwent follow-up CEUS examinations between February 2008 and February 2020. Twelve out of 364 patients underwent renal surgery without follow-up CEUS. The progression rate of Bosniak 2F renal lesions detected by CEUS accounted for 7.1% (8/112 patients) after a mean of 12.9 months. The first follow-up CEUS revealed 75% of progressions (6/8), the remaining 25% (2/8) of progressions were detected during second follow-up CEUS. Underlying clear-cell renal cell carcinoma was histopathologically validated in 5/8 progressive complex cystic renal lesions. Stable sonomorphologic features were observed in 92.1% (104/112 patients). CEUS depicts a promising diagnostic imaging modality in the diagnostic work-up and follow-up of complex renal cystic lesions at higher spatial and temporal resolutions than CT or MRI. Its excellent safety profile, its easy and repeatable accessibility, and low financial costs render CEUS an attractive and powerful alternative imaging tool for monitoring complex renal cystic lesions.

## 1. Introduction

Renal cell carcinoma (RCC) comprises approximately 3% of all cancer entities and depicts the urological cancer with the highest mortality rates with >40% [1]. Almost every third patient shows metastasizing disease when RCC is diagnosed. Due to advancing imaging techniques, the detection rates of RCC have substantially improved during the last decades. Nevertheless, a relevant amount of incidentally detected renal lesions is left indeterminate and further diagnostics are required. In approximately 8% of cases, RCCs present as complex cystic lesions. Cystic renal lesions show an increasing incidence with older age and appear in over half of patients >50 years [2]. Up to date, incidental renal lesions are more frequently found, particularly because of more elaborate cross-sectional imaging, computed tomography (CT) and magnetic resonance imaging (MRI). The majority of these renal lesions are uncomplicated cysts of benign origin [3].

However, associated complicating morphologic features, including mural thickening, spots of calcifications, nodularity, septations, and contrast-enhancement may be seen in up to 10% of cases in incidentally found renal lesions. Thus, those lesions must be considered indeterminate and potentially malignant. Consequently, for those cases, consistent follow-up, or even surgical and histopathological evaluation are mandatory.

**Figure 1:**
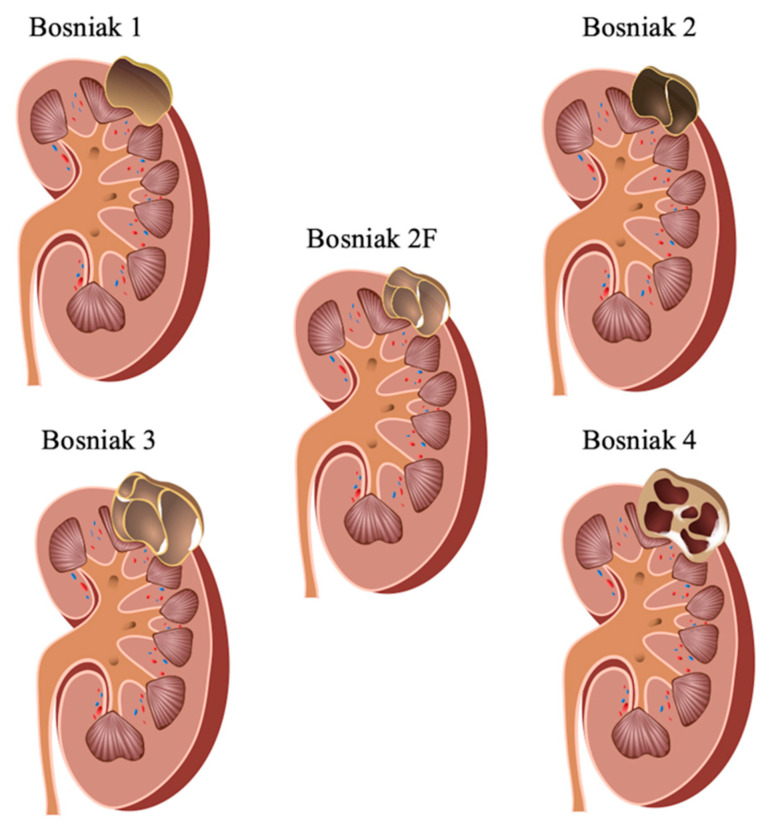
Illustration of Bosniak classification and the corresponding morphological features. Bosniak 1—hairpin-thin cystic walls, well-defined; Bosniak 2—septa <1 mm, fine septal calcifications, no contrast-enhancement/discrete septal contrast-enhancement; Bosniak 2F—multiply septated, thickened cystic walls, thin/thick calcifications, discrete mural/septal contrast-enhancement; Bosniak 3—homogeneous/irregular thickening of septa/wall, irregular calcifications, increasing septal contrast-enhancement; Bosniak 4—features of Bosniak 3 + solid components, irregular solid contrast-enhancing components.

The classification system by M.A. Bosniak has simplified radiologists to classify renal lesions and help assess the likelihood of underlying benignity and malignancy by means of CT criteria since its introduction in 1986 [4] (Figure 1).

The initial Bosniak classification comprised four subtypes (1–4) and was later complemented by category 2F (“F” = follow-up) [3,4,5,6]. According to the recent Bosniak classification, renal cysts may thus be categorized into five different subtypes depending on cyst morphology and contrast enhancement characteristics (1, 2, 2F, 3, 4, “F” = follow-up). Bosniak 1 and 2 subtypes are associated with malignancy rates of almost 0%. Bosniak 2F depicts complex cystic lesions with complexity between Bosniak 2 and Bosniak 3 lesions and may have malignant potential in up to 5% of the cases. The Bosniak 2F category allows for non-invasive monitoring of complex renal cystic lesions. However, up to date, it is unclear how long Bosniak 2F lesions need to be monitored before intervention is necessary. The time to progression and the associated probability is uncertain in literature, but up to 4 years for complex cystic Bosniak 2F lesions has been suggested [7]. Several studies reported a low progression rate but with a high associated malignancy rate after observed progression [8,9]. Bosniak 3 and 4 feature malignancy in ~50% and ~100% of the cases, respectively [10,11]. Overlapping morphologic gaps between benignity and malignancy of renal cystic lesions further challenge diagnostic imaging. Noteworthy, determining an adequate therapeutic treatment option is substantially dictated by precise categorizing renal cystic lesions according to the Bosniak classification system.

A pivotal morphologic characteristic that may allow for differentiating benign from malignant entity depicts contrast-enhancement, which can be registered by using cross-sectional imaging modalities, such as CT or MRI. Extensive evaluation before being performed is necessary for CT and MRI because of ionizing radiation, potential allergic predisposition to iodinated contrast agent, and thyroidal and renal affections in case of CT. In the case of MRI, potential allergic reactions to MRI-contrast agents or in-situ metallic medical devices, e.g., implantable cardioverter defibrillator (ICD) must be evaluated. Conventional ultrasound, comprising native B-mode and Color Doppler, is not capable to visualize microperfusion and thereby contrast enhancement. Those shortcomings of conventional ultrasound are resolved by contrast-enhanced ultrasound (CEUS), which is feasible to visualized microperfusion of tissue and tumors at higher spatial and temporal resolutions juxtaposed to CT and MRI [12]. Contrast-enhanced ultrasound has previously shown high diagnostic performance in imaging of the kidneys [13,14,15,16]. Moreover, it was shown that CEUS helped to distinguish between benign and malignant renal lesions and could shed more light on indeterminate renal lesions. Contrast-enhanced ultrasound depicts an accurate diagnostic instrument, which enables to even track single microbubbles within septa or cystic walls [17]. The visualization of the microcirculation helps to adequately categorize renal cystic lesions into Bosniak subtypes [18]. Of utmost importance, CEUS is readily accessible and repeatable, comparably inexpensive and has an excellent safety profile [19].

The purpose of the present study was to assess the evolution of Bosniak 2F renal cystic lesions, in particular the rate of progression to malignancy, during follow-up CEUS examinations.

## 2. Results

Between February 2008 and February 2020, 364 patients underwent renal CEUS, which detected 381 Bosniak 2F renal cystic lesions in total. One-hundred-and-twelve out of 381 (29.4%) patients with Bosniak 2F lesions were re-assessed during follow-up CEUS scans. There was a male predominance in the follow-up cohort (male:female ratio = 75:37, approximately 2:1); twelve out of 364 (3.3%) patients with Bosniak 2F lesions underwent renal surgery without follow-up CEUS. One-hundred-and-twenty out of 364 (33.0%) patients were included in the present retrospective single-center study. Four out of 108 (3.7%) of the included patients showed bilateral Bosniak 2F lesions. Sixty four out of 112 (57.1%) Bosniak 2F lesions were located in the left kidney, 45/112 Bosniak 2F (40.2%) were located in the right kidney, and 3/112 (2.7%) Bosniak 2F lesions were detected in transplanted kidneys. The mean size of the Bosniak 2F lesions was 3.7 cm (SD ± 2.8 cm); median size was 3.0 cm (IQR: 1.5–5.0 cm).

In total, 260 follow-up CEUS (fu-CEUS) examinations were conducted for monitoring 112 Bosniak 2F lesions.

The mean age of the patients who underwent follow-up CEUS scans accounted to 60.7 years (SD ± 11.7 years), median age of 61 years (IQR: 29–82 years) at initial CEUS. All 112 patients underwent first follow-up CEUS after a mean of 12.2 months (SD ± 2.6 months). Fifty-two out of 112 (46.4%) of the included patients underwent second follow-up CEUS with a mean interval of 9.3 months (SD ± 7.1 months) between the first and the second follow-up CEUS. Further fu-CEUS examinations were as follows: 3rd fu-CEUS in 35/112 patients (31.3%) after a mean of 8.8 months (SD ± 4.4 months), 4th fu-CEUS in 23/112 (20.5%) after a mean of 12.0 months (SD ± 6.8 months), 5th fu-CEUS in 14/112 (12.5%) after a mean of 11.5 months (SD ± 3.7 months), 6th fu-CEUS in 9/112 (8.0%) after a mean of 8.0 months (SD ± 5.8 months), 7th fu-CEUS in 5/112 (4.5%) after a mean of 12.5 months (SD ± 7.7 months), 8th fu-CEUS in 4/112 (3.6%) after a mean of 10.7 months (SD ± 6.1 months), 9th fu-CEUS in 2/112 after a mean of 8.0 months (SD ± 2.8 months), 10th fu-CEUS in 2/112 (1.8%) after a mean of 5 months (SD ± 2.8 months), and 11th and 12th fu-CEUS in 1/112 (0.9%) after 5 months respectively.

According to the Bosniak classification, progressive Bosniak lesions were detected in 8/112 (7.1%) patients in fu-CEUS with a preponderance in men (male:female ratio for progression = 3:1) (Table 1). Stable Bosniak 2F lesions were described during fu-CEUS in 104/112 (92.9%) patients. The mean time to progression of Bosniak 2F lesions from initial CEUS was 12.9 months (SD ± 12.7 months), median time to progression was 9.5 months (IQR: 3–43 months). First fu-CEUS revealed progressive renal lesions in 6/8 (75%) patients, whereas progression was registered in the 2nd fu-CEUS in 2/8 (25%).

**Figure 2:**
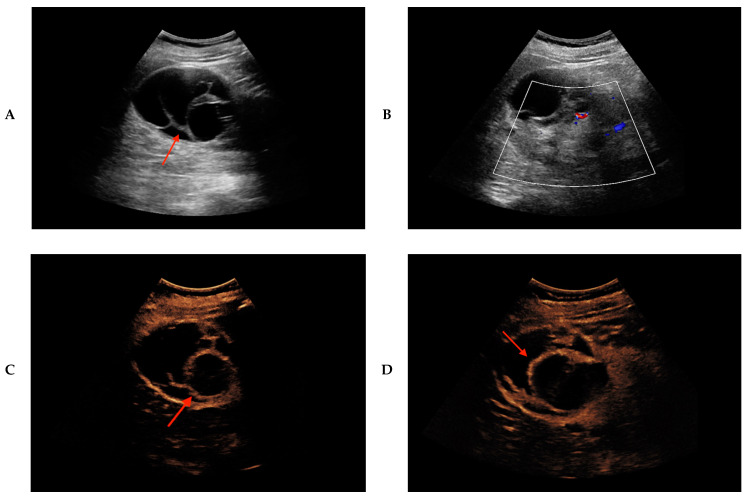

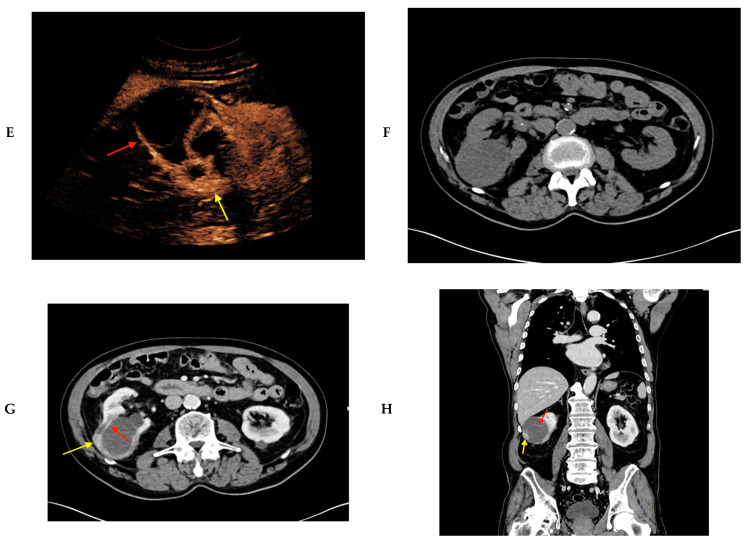
Progression of Bosniak 2F renal cystic lesion to Bosniak 4 category revealed by CEUS in a 67-year-old male patient. Partial nephrectomy and histopathology revealed underlying clear cell renal cell carcinoma. (**A**) Septated cystic renal lesion (red arrow) of the right kidney is registered by native B-mode ultrasound, in keeping with Bosniak Type 2F. (**B**) No hyperperfusion is detected in Color Doppler ultrasound. (**C**) CEUS reveals bright contrast-enhancement of the septa (red arrow). (**D**) Follow-up CEUS after 14 months shows increased contrast-enhancing of the thickened septa (red arrow) of the cystic lesion, thus classified as Bosniak Type 3. (**E**) Besides bright septal contrast-enhancement (red arrow), 28 months follow-up CEUS further reveals additional nodular contrast-enhancement (yellow arrow) of the cystic lesion, hence classified as Bosniak Type 4. (**F**) No calcification is detected in the renal cystic lesion in the corresponding native CT, axial reformation. (**G**) Septal and nodular contrast-enhancement (red and yellow arrow, respectively) of the renal cystic lesion in the corresponding contrast-enhanced CT, venous phase, axial reformation. (**H**) Septal and nodular contrast-enhancement (red and yellow arrow, respectively) of the renal cystic lesion in the corresponding contrast-enhanced CT, coronal reformation, venous phase.

Progression of Bosniak 2F lesions and upward classification to Bosniak 3 was detected in 7/8 (88%) patients. In one (13%) patient (Patient 2), progression and upward classification to Bosniak 3 and finally to Bosniak 4 was detected. Six patients in whom progression from initial Bosniak 2F lesion was observed during fu-CEUS underwent (partial) nephrectomy, and histopathological analysis could finally reveal the underlying tumor entity (Patients 1, 2, 3, 4, 5, 7), of whom 5/6 (83%) showed progression to Bosniak 3 and 1/6 (17%) to Bosniak 4 category (Figure 2).

Histopathology revealed clear-cell renal cell carcinoma (ccRCC) in five patients. Despite observed progression and upward categorization from Bosniak 2F to Bosniak 3 during fu-CEUS and description of a suspicious contrast-enhancing renal cystic lesion in keeping with Bosniak 4 lesion in one patient (Patient 5), histopathology unveiled benign renal leiomyoma. Patient 3 did not undergo bioptical validation nor renal surgery, although fu-CEUS 10 months after initial CEUS depicted increased intraseptal and peripheral contrast-enhancement could be visualized in keeping with Bosniak 3; corresponding CT 10 months after initial CEUS showed nodular contrast enhancement and partially solid components of the renal lesion; thus, findings from both follow-up imaging modalities, CEUS and CT, implied malignancy. The Bosniak 2F lesion in Patient 6 showed progression in 6-month fu-CEUS to Bosniak 3 category due to increased septal and nodular contrast enhancement of the lesion. In the corresponding MRI at 6-months after initial CEUS, no contrast-enhancing septa could be observed, indicating hemorrhagic cyst. No bioptical validation nor renal surgery was followed in Patient 6.

In 3/112 (2,7%) patients, regression from initial Bosniak 2F category could be observed during first fu-CEUS, twice regression and subsequent downward classification to Bosniak 2 and once regression and downward classification to Bosniak 1.

In 12 patients (12/381, 3.1%), immediate renal biopsy or surgical resection was conducted without prior fu-CEUS (Table 2). In 4/12 (33%) patients, (partial) nephrectomy was performed, histopathology revealed ccRCC in 2/4 patients, and papillary RCC (pRCC) in the remaining 2/4 patients. Malignancy was excluded in the remaining 8/12 patients.

## 3. Discussion

Close collaboration between radiologists and urologists is mandatory for classifying complex renal cystic lesions and directing appropriate treatment. Bosniak 2F cystic lesions depict a rare subtype that morphologically varies, may be moderately to more complex, and can progress to higher-category lesions, Bosniak 3 and 4 [20]. To date, extensive (sono-)morphological evaluation with histopathological correlation of Bosniak 2F lesions is sparse. Hence, the Bosniak 2F subtype still depicts a tremendous challenge for physicians regarding both adequate diagnostic and treatment. According to recent literature active surveillance of Bosniak 2F, cysts help to identify lesions that need surgical intervention [21].

Recommendations for follow-up of Bosniak 2F show a broad variety in the literature and are a matter of debate up until today. According to the Canadian Urological Association (CUA), follow-up imaging by contrast-enhanced CT or MRI every 6 months in the first year upon first detection, following annual follow-up for at least 5 years, is recommended [22]. It is further stated that CEUS may help as an adjunct in visualizing and assessing septations, wall texture and contrast-enhancement. According to the American College of Radiology (ACR), incidentally detected Bosniak 2F cystic lesions are recommended to be followed-up either by CT or MRI at 6 and 12 months, and then annually for 5 years [23].

The role of MRI for Bosniak classification was subject in several clinical trials. Non-ionizing MRI was reported to be superior to CT in detecting septations, mural/septal thickness and contrast-enhancement of solid parts of complex renal lesions and may thus even allow for differentiating between histologic subtypes [24,25]. In a retrospective analysis, sensitivity and specificity of MRI for diagnosing renal clear-cell carcinoma was 92% and 83%, and for diagnosing renal papillary carcinoma it was 80% and 94%, respectively [26]. Accurate visualization of cystic septations was described to be the main aspect for category migration [27]. Hence, due to its superior soft tissue and contrast resolution compared to CT, MRI surveillance of complex renal cystic lesions may lead to migration of Bosniak categories, thereby directing appropriate therapeutical management of patients. The characterization and categorization of complex renal cystic lesions by MRI was recently complemented in Bosniak Classification in 2019 [28]. Unfavorable for MRI is its limited availability; its comparably high financial costs; potential limitations due to claustrophobia; kidney failure, which limits administration of contrast agents; and in-situ metallic medical devices like pacemakers or allergic predisposition of the patient to MR-contrast agents. Moreover, the recently reported deposition of gadolinium-based contrast agents (GBCA) in the basal nuclei, globus pallidus and dentate nucleus, need to be kept in mind, particularly in case of younger patients [29]. So far, no evident clinical affection due to the GBCA deposition in the central nervous system has been described.

By using the random Brownian motion of water molecules, tissue characteristics can further be evaluated. Diffusion-weighted MRI was previously shown to be helpful for characterizing the entity of renal lesions [30,31]. Elevated signals in diffusion-weighted imaging (DWI) and corresponding reduced apparent diffusion coefficient (ADC) signals were described to be found in malignant tumors compared to benign tumors [32,33,34]. By applying a cut-off ADC of 1.66 × 10^−3^ mm/s^2^, a diagnostic sensitivity and specificity of 90% and 83% of DW-MRI for distinguishing RCC and renal oncocytoma was described [35].

Computed tomography (CT) depicts an imaging modality with high diagnostic performance for assessing malignant renal lesions. Pooled sensitivity, specificity, positive likelihood ratio and negative likelihood ratio for evaluating renal lesions by CT accounted for 0.90, 0.85, 5.00 and 0.15, respectively within the frame of a meta-analysis [36]. Pooled sensitivity and specificity by applying the Bosniak classification system by CT was described to be 89.6% and 65.1% [37].

In case of follow-up by means of CT scans, despite advancing radiation-sparing technologies, one has to consider the cumulative exposure to ionizing radiation [38], which relatively increases the risk of radiation-related cancer [39], considering also that most Bosniak 2F lesions stay stable and are of benign entity. Using a microsimulation model revealed that radiation-related cancer risk due to CT surveillance of Bosniak 2F lesions is marginal on the basis of a mean age of 64 years [40]. However, due to the increasing application of CT scans, these radiation-induced risks would plausibly be elevated in younger patients.

In case of indeterminate and incidentally found renal cystic lesions, CEUS previously proved to be a valid diagnostic tool to classify those lesions and was reported to have equivalent diagnostic accuracy compared to CT [13,41,42]. A meta-analysis of 11 included clinical studies revealed pooled, sensitivity, specificity, positive likelihood ratio, and negative likelihood ratio of 0.95, 0.79, 4.39, and 0.10, respectively, of CEUS for analyzing malignant renal tumors [36].

In a meta-analysis comprising 17 clinical studies, CEUS showed a higher diagnostic sensitivity, but less specificity for assessing complex cystic renal masses compared to MRI [43]. By applying CEUS-MRI image fusion, the advantages of both imaging modalities can be combined: cross-sectional imaging in a real-time manner [44,45]. Without hesitation and time delay, incidentally, found renal cystic lesions may immediately be further assessed by performing CEUS, hence sparing patient distress, and otherwise applying subsequent CT or MRI scan for further analysis of the renal lesion [46,47]. Of note, CEUS may easily and repeatably be applied without comparable prior thorough evaluation—likewise in the case of CT or MRI. Due to its excellent safety profile [19], CEUS may be safely performed on patients with (chronic) kidney failure, disbalances of the thyroid gland or allergic predispositions to iodinated or gadolinium-derived contrast-agents. Furthermore, recent studies reported the safe application of CEUS during pregnancy [48,49] when more elaborate (contrast-enhanced) imaging modalities are only restrictively performed in order to safeguard fetal and maternal well-being. Of note, the cost-effectiveness of CEUS for evaluating and categorizing indeterminate renal cystic lesions in juxtaposition with MRI was recently described [50]. Previous findings from the literature, the findings from our present study, and the fact that CEUS has been approved by the U.S. Food and Drug Administration (FDA) in 2016, may direct future clinical decision making in terms of complex renal cystic lesions, in particular the borderline subtype Bosniak 2F. Neither of the leading societies have recommend CEUS as primary imaging modality so far. Moreover, in the frame of latest Bosniak classification of 2019, the role of CEUS is not fully established yet [28].

Several prior clinical studies investigated the evolution of Bosniak 2F cystic lesions during follow-ups mainly by cross-sectional imaging modalities, CT and MRI [51]. Bosniak 2F cystic lesions showed progression to Bosniak 3 category in 14 of 201 included patients (7%), with a mean time of progression of 11 months [52]. Typically, upward classification was reported to occur within the first 2 years [53]. In a multicentric study, 9/69 (13%) Bosniak 2F cystic lesions progressed during follow-up CT examinations, of whom 50% revealed to be malignant, leading to a malignancy rate of 25% for Bosniak 2F lesions in this cohort [54]. A recent retrospective study, assessing morphology of Bosniak 2F lesions in 143 patients by CT and MRI, could demonstrate a progression rate of 4.6% of Bosniak 2F lesions with a mean time to progression of 20 months [8]. In a prospective single-center study, CEUS showed higher sensitivity and specificity compared to MRI in the context of follow-up of Bosniak 2F renal cystic lesions [55]. Similar to previous results from the literature, the rate of progressed Bosniak 2F lesions accounted for 7.1% during fu-CEUS after a mean time of 12.9 months in the present study. Seventy-five percent (6/8) of progressive Bosniak 2F cystic lesions were detected within the 1st fu-CEUS, the remaining 25% (2/8) of progressive Bosniak 2F cystic lesions were observed in the 2nd fu-CEUS. Our findings are compatible with previously studies that demonstrated that progression characteristically develops within the first 2 years after first detection. In 7/8 cases, Bosniak 2F lesions progressed to Bosniak 3 category, in 4/7 of whom, histopathology revealed clear-cell RCC. One patient (patient 5) in whom a 1.6 cm measuring progressive lesion was upwardly categorized to Bosniak 3 subtype by CEUS, further, which even was upwardly classified to Bosniak 4 category due to MRI findings, underwent partial nephrectomy, and histopathology finally revealed leiomyoma. Prior findings already demonstrated the diagnostic challenge to distinguish small-sized benign renal tumors, including leiomyoma, especially from small RCCs [56,57]. Two patients in whom progression to Bosniak 3 category was observed, declined urological resection, thus histopathological correlation is lacking. In one patient (patient 2), progressive evolution from Bosniak 2F to Bosniak 3 and then to Bosniak 4 was observed during fu-CEUS over 28 months, whereas findings from corresponding contrast-enhanced CT classified the complex cystic lesion to Bosniak 4 after 8 months. Due to compromising comorbidities, watch-and-wait strategy was first conducted until after 28 months, the patient underwent partial nephrectomy, and histopathology confirmed clear-cell RCC. In line with the literature, the majority of Bosniak 2F lesions featured stable sonomorphology (92,9%); 3.1% (12/381) patients were not monitored by means of fu-CEUS, but instead underwent immediate (partial) renal resection. Benignity was histopathologically confirmed in 8/12 (75%). Underlying papillary RCC and clear-cell RCC was validated in 2/4 (50%) and in 2/4 (50%), respectively.

The majority of cases that showed morphological progression of the Bosniak 2F cystic lesion and revealed to be of malignant origin, depict low-stage RCC and are associated with relatively good prognoses due to the low rate of metastasizing disease [58,59]. In line with the recent state of knowledge, our findings show that follow-up of Bosniak 2F complex cystic lesions on the one hand is justified, and on the other hand can precisely be conducted by means of CEUS.

Characteristically, RCCs usually are hypervascularized, therefore, featuring broad contrast-enhancement in diagnostic imaging. To date, CT and MRI have been the primarily recommended imaging modalities for the diagnostic work-up and follow-up of complex renal cystic lesions. Some societies recommend CEUS as an additional imaging instrument. The fact that CEUS allows for visualizing microperfusion of (tumorous) tissue at even higher spatial and temporal resolutions and in a real-time manner compared to more elaborate CT and MRI renders CEUS as a powerful and promising tool. Of course, cross-sectional imaging cannot be replaced by CEUS in terms of staging.

The present study has some limitations. The study was conducted retrospectively. A relevant proportion of Bosniak 2F was excluded due to lacking follow-up examinations, which might have prompted selection bias. Contrast-enhanced ultrasound was performed in every single patient by the same experienced consultant radiologist (EFSUMB level 3) at our University Hospital. The further work-up and follow-up of the patients was predominantly determined by the referring Department of Urology, thus explaining the heterogeneity of follow-up timepoints.

## 4. Materials and Methods

The local institutional ethical committee of the institutional review board (Ethics Committee, Medical Faculty, Ludwig-Maximilians-University Munich) approved the present retrospective monocenter study (Project No. 17-087, date of approval: 14 March 2017). All study data were collected and retrieved respecting the principles expressed in the Declaration of Helsinki/Edinburgh 2002. Prior to every CEUS examination, oral and written informed consent were obtained by all patients. The procedure of CEUS, potential risks and unlikely complications have been thoroughly explained. One single skilled consultant radiologist with over 15 years of experience (EFSUMB level 3) performed all CEUS scans. All ultrasonographic examinations comprised native B-mode, Color Doppler and CEUS. In every case up-to-date, high-end ultrasound devices (GE Healthcare LOGIQ L9, Chicago, IL, USA—transducer: C5-1; Siemens Ultrasound Sequoia, ACUSON Sequoia, Mountain View, CA, USA—transducer: C4-1; Philips Ultrasound iU22, EPIQ 7, Seattle, WA, USA—ransducer: C9-2) with appropriate CEUS protocols and at low mechanical index (<0.2) to prevent early destruction of microbubbles. Intravenous application of 1.0–2.4 mL of SonoVue^®^, a second-generation blood pool contrast agent (Bracco, Milan, Italy) and an additional 5–10 mL sterile 0.9% sodium chloride was done in every single CEUS examination. During the entire study, no adverse side effects after administration of SonoVue^®^ could be registered. CEUS scans were all successfully conducted; sufficient image quality allowed for adequate evaluation of the sonomorphology of renal lesions of interest. Morphological characteristics that were evaluated comprised: echogenicity, size, and shape. Vascularization was analyzed by Color Doppler and CEUS. During CEUS scans, the vascular phases included cortical phase (8–35 s after i.v. administration of contrast agent), corticomedullary phase (36–120 s i.v. administration of contrast agent) and late phase (>120 s until vanishing of the microbubbles).

Stored cine-loops of the entire included patient cohort were retrospectively evaluated. All patient data and imaging files were stored and retrieved from the institutional picture archiving and communication system (PACS).

**Figure 3:**
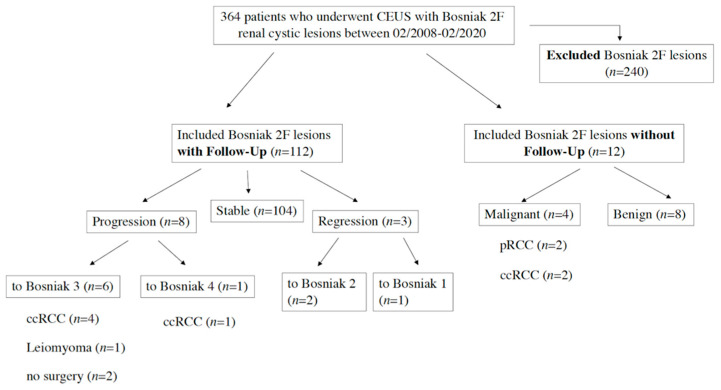
Flowchart illustrating included and excluded patients with Bosniak 2F renal cystic lesions. ccRCC—clear-cell renal cell carcinoma, pRCC—papillary renal cell carcinoma.

Between February 2008 and February 2020, 364 patients underwent renal CEUS, which detected 381 Bosniak 2F renal cystic lesions in total (Figure 3). One-hundred-and-twelve out of 364 (30.8%) patients with Bosniak 2F lesions underwent follow-up CEUS scans. Twelve out of 364 (3.3%) patients with Bosniak 2F lesions underwent renal biopsy or renal surgery without follow-up CEUS. Two-hundred-and-forty patients were excluded who either did not undergo follow-up CEUS or renal biopsy/renal surgery was not conducted.

Renal surgery, either partial or complete nephrectomy, was performed in the local Department of Urology. The histopathological analysis was performed in collaboration with the local Institute of Pathology. Histopathological results were used as the diagnostic reference standard.

## 5. Conclusions

To our knowledge, the present retrospective study comprises the largest cohort of patients with Bosniak 2F complex renal cystic lesions, which were monitored in follow-up examinations by CEUS. The fact that Bosniak 2F lesions seldomly metastasize and develop local infiltrative growth justifies initial watch-and-wait policy. In case of progressive complex renal cystic lesion and thus upward categorization according to the Bosniak classification, bioptical validation or renal surgery need to be evaluated. Its easily and repeatably accessibility, its excellent safety profile, its low financial costs, and its capability to visualize tumor microperfusion at higher spatial and temporal resolutions compared to CT and MRI, renders CEUS a powerful and promising imaging modality in the diagnostic work-up and follow-up of complex renal cystic lesions, particularly for Bosniak 2F category, even in younger patients.

## Figures and Tables

**Table 1:** Patients with initial Bosniak 2F lesions, which progressed during follow-up CEUS.

Patients	Age	Sex	Size (cm)	Location	Native B-mode	Vascularization (CD)	CEUS	CT/MRI	Treatment	Histopathology
1	53	M	1.0	L	Cystic	-	septated, no contrast enhancement (Bosniak 2F)	CT: thin intracystic septa, (Bosniak 2F)	Partial nephrectomy	ccRCC
					FU (11 mo): Increasing size: 1.8 cm	-	no contrast enhancement (Bosniak 3)			
2	67	M	7.0	R	Septated cystic lesion	-	CEUS: Septal contrast enhancement (Bosniak 2F)	CT (8 mo): Contrast enhancing septated cystic lesion (Bosniak 4)	Partial nephrectomy	Well-differentiated, highly-regressive ccRCC
					FU (3 mo): Same morphology	-	Bosniak 2F			
					FU(14 mo)	-	Progressive and contrast enhancing septa (Bosniak 3)			
					FU(21 mo)	-	Same morphology (Bosniak 3)			
					FU(28 mo)	-	Progressive and contrast enhancing septa (Bosniak 4)			
3	55	M	2.0	L	Cystic	-	Visualization of intracystic septa; septal contrast enhancement (Bosniak 2F)	CT (12 mo): Nodular contras enhancement, partially solid	-	-
					FU(10 mo): partial solid cystic lesion	-	Intraseptal + marginal contrast enhancements (Bosniak 3)			
4	56	M	1.5	1	Septated cystic	-	Septal contrast enhancement (Bosniak 2F)	CT (3 mo): Hypodenes, septated renal mass, nodular contrast-enhancing (Bosniak 3)	Partial nephrectomy	Moderately differentiated ccRCC
					FU(2 mo): increasing size, 3.5 cm	-	Septal and nodular contrast enhancement (Bosniak 3)	-	-	-
5	66	F	1.6	L	Cystic	-	Septated, septal contrast-enhancement (Bosniak 2F)	MRI (6 mo): Contrast-enhancing cystic lesion, delayed wash-out (Bosniak 4)	Partial nephrectomy	Leiomyoma
					FU(6 mo)	-	Bosniak 3			
6	69	M	2.4	R	Cystic	-	Visualization of intracystic septa, septal contrast-enhancement (Bosniak 2F)	MRI (6 mo): Wall thickened renal cystic lesion, no contrast enhancement (hemorrhagic cyst)	-	-
					FU(6 mo)	-	Increasing septal and nodular contrast-enhancement (Bosniak 3)			
7	79	M	5.0	R	Cystic	-	Early and bright contrast-enhancement (Bosniak 2F)	CT: Inhomogeneously contrast-enhancing cystic lesion (Bosniak 3) MRI (8 mo): Inhomogeneously contrast-enhancing cystic lesion (Bosniak 3)	Nephrectomy	Moderately differentiated ccRCC
					FU (30 mo)	-	Increasing contrast-enhancement of the cystic lesion (Bosniak 3)			
8	79	F	1.7	L	Cystic	-	Intracystic septa, intraseptal and marginal contrast-enhancement (Bosniak 2F)	-	Partial nephrectomy	Well-differentiated ccRCC
					FU (12 mo)		Increasing contrast-enhancement of the septa (Bosniak 3)	-	-	-

ccRCC—clear-cell renal cell carcinoma, CD—Color Doppler, CEUS—contrast-enhanced ultrasound, CT—computed tomography, F—female, FU—follow-up, L—left, M—male, mo—months, MRI—magnetic resonance imaging, pRCC—papillary renal cell carcinoma, R—right.

**Table 2:** Patients with Bosniak 2F lesions who underwent renal surgery without follow-up CEUS.

Patients	Age	Sex	Size (cm)	Location	Native B-mode	Vascularization (CD)	CEUS	CT/MRI	Treatment	Histopathology
1	45	M	7.0	R	Partial solid	-	No enhancement (Bosniak 2F)	-	Partial nephrectomy	Well-differentiated ccRCC
2	67	M	1.0	R	Partial solid cystic	-	No enhancement (Bosniak 2F)	-	Nephrectomy	Well-differentiated pRCC, WHO Type I
3	59	M	1.0	L	Cystic	-	Moderate contrast enhancement of the cystic lesion (Bosniak 2F)	-	Partial nephrectomy	Well-differentiated ccRCC
4	70	M	2.5	R	Cystic	-	Early contrast-enhancement, venous wash-out (Bosniak2F)	-	Partial nephrectomy	pRCC, WHO Type II

ccRCC—clear-cell renal cell carcinoma, CD—Color Doppler, CEUS—contrast-enhanced ultrasound, CT—computed tomography, pRCC—papillary renal cell carcinoma, L—left, M—male, MRI—magnetic resonance imaging, R—right.
